# LncRNA SNHG4 promotes osteosarcoma proliferation and migration by sponging miR‐377‐3p

**DOI:** 10.1002/mgg3.2113

**Published:** 2022-11-28

Huang, Y. F., Lu, L., Shen, H. L., & Lu, X. X. (2020). LncRNA SNHG4 promotes osteosarcoma proliferation and migration by sponging miR‐377‐3p. *Molecular Genetics & Genomic Medicine*, *8*(8), e1349. https://doi.org/10.1002/mgg3.1349

The above article published online on 14 June 2020 in Wiley Online Library (www.wileyonlinelibrary.com), has been retracted by agreement between the Editor in Chief Prof. Suzanne Hart and John Wiley & Sons. The retraction has been agreed due to concerns raised about image manipulation. The journal team has investigated and determined that the image manipulation undermines the reliability of the data presented and the article's conclusions.

